# Surgery-Induced Changes and Early Recovery of Hip-Muscle Strength, Leg-Press Power, and Functional Performance after Fast-Track Total Hip Arthroplasty: A Prospective Cohort Study

**DOI:** 10.1371/journal.pone.0062109

**Published:** 2013-04-16

**Authors:** Bente Holm, Kristian Thorborg, Henrik Husted, Henrik Kehlet, Thomas Bandholm

**Affiliations:** 1 The Lundbeck Centre for Fast-track Hip and Knee Arthroplasty, Copenhagen University Hospital, Hvidovre, Denmark; 2 Department of Physical Therapy, Physical Medicine and Rehabilitation Research – Copenhagen (PMR-C), Copenhagen University Hospital, Hvidovre, Denmark; 3 Arthroscopic Centre Amager, Copenhagen University Hospital, Amager, Copenhagen, Denmark; 4 Department of Orthopedic Surgery, Copenhagen University Hospital, Hvidovre, Denmark; 5 Section for Surgical Pathophysiology, Copenhagen University Hospital, Rigshospitalet, Copenhagen, Denmark; 6 Clinical Research Centre, Copenhagen University Hospital, Hvidovre, Copenhagen, Denmark; The University of Queensland, Australia

## Abstract

**Background:**

By measuring very early changes in muscle strength and functional performance after fast-track total hip arthroplasty (THA), post-operative rehabilitation, introduced soon after surgery, can be designed to specifically target identified deficits.

**Objective(s):**

Firstly, to quantify changes (compared to pre-operative values) in hip muscle strength, leg-press power, and functional performance in the first week after THA, and secondly, to explore relationships between the muscle strength changes, and changes in hip pain, systemic inflammation, and thigh swelling.

**Design:**

Prospective, cohort study.

**Setting:**

Convenience sample of patients receiving a THA at Copenhagen University Hospital, Hvidovre, Denmark, between March and December 2011.

**Participants:**

Thirty-five patients (65.9±7.2 years) undergoing THA.

**Main outcome measures:**

Hip muscle strength, leg-press power, performance-based function, and self-reported disability were determined prior to, and 2 and 8 days after, THA (Day 2 and 8, respectively). Hip pain, thigh swelling, and C-Reactive Protein were also determined.

**Results:**

Five patients were lost to follow-up. Hip muscle strength and leg press power were substantially reduced at Day 2 (range of reductions: 41–58%, *P*<0.001), but less pronounced at Day 8 (range of reductions: 23–31%, *P*<0.017). Self-reported symptoms and function (HOOS: Pain, Symptoms, and ADL) improved at Day 8 (*P*<0.014). Changes in hip pain, C-Reactive Protein, and thigh swelling were not related to the muscle strength and power losses.

**Conclusion(s):**

Hip muscle strength and leg-press power decreased substantially in the first week after THA – especially at Day 2 – with some recovery at Day 8. The muscle strength loss and power loss were not related to changes in hip pain, systemic inflammation, or thigh swelling. In contrast, self-reported symptoms and function improved. These data on surgery-induced changes in muscle strength may help design impairment-directed, post-operative rehabilitation to be introduced soon after surgery.

**Trial Registration:**

ClinicalTrials.gov NCT01246674.

## Introduction

Total hip arthroplasty (THA) is commonly performed to relieve pain and improve functional performance in patients with end-stage hip osteoarthritis [1]. In many places, it is followed by out-patient physical rehabilitation, with the rehabilitation components sometimes differing between programs [2], [3]. However, despite undergoing physical rehabilitation after THA, reduced muscle strength and functional performance have been reported at various time points when compared to the preoperative level [4]–[12], which may already have been reduced due to end-stage osteoarthritis [4], [5], [8], [10]–[15].

Muscle strength losses have been reported for several muscle groups in the operated leg after THA, including those associated with hip abduction [4], [14]–[19], adduction [4], [5], flexion [4], [5], [8], [14]–[19], and extension [4], [5], [8], [14]–[19], as well as knee extension [4], [5], [12], [14], [19] and flexion [4]. With a few exceptions [20]–[22], these persisting multi-level strength deficits have primarily been reported in the later post-operative phase, that is, after the completion of out-patient physical rehabilitation, which is typically not well documented with respect to, for example, the applied strength training exercises (choice of exercises, intensity, volume, etc.) [23]. Hence, it is difficult to judge if persisting muscle strength deficits after THA appear to have been sufficiently targeted during the physical rehabilitation programs. In order to do so, early strength deficits induced by surgery need to be quantified, in order to design a specific, impairment-directed, postoperative rehabilitation program for implementation soon after surgery. This approach is an important initial step in the fast-track/enhanced recovery program methodology [24], [25], which combines evidence from different peri-operative uni-modal interventions, including post-operative physical rehabilitation [23].

With respect to the mechanisms underlying the early loss of muscle strength after THA, failure of the central nervous system to activate muscles close to the operated hip joint due to intra-articular swelling may be involved [26]. This is also seen after total knee arthroplasty, and is commonly referred to as arthrogenic muscle inhibition [27], [28]. Besides swelling, arthrogenic muscle inhibition has also been linked to inflammation, pain, joint laxity, and structural joint damage [28] – conditions that are known to occur soon after THA. Over time, atrophy of several muscles around the hip and knee joints in the operated leg seems to develop [29], [30], which further contributes to the loss of muscle strength.

The primary objective of this study was to quantify changes (compared to preoperative values) in hip muscle strength, leg-press power, and functional performance within the first week after THA – before the start of out-patient physical rehabilitation. The secondary objective was to explore relationships between the muscle strength changes, and changes in hip pain, systemic inflammation, and thigh swelling. We hypothesized that muscle strength, power, and performance-based function would be substantially reduced in the first week after THA, compared to preoperative values.

## Methods

### Ethics statement

All patients were provided written information about the procedures of the study, and informed consent was obtained in strict accordance with the Declaration of Helsinki. The Ethics Committee in Copenhagen approved the study. (H-4-2010-FSP). ClinicalTrials.Gov-identifier: NCTO1246674, http://clinicaltrials.gov/ct2/show/NCT01246674.

### Design

Patients, scheduled for a primary unilateral THA, were evaluated one week before surgery (Pre-surgery), one day post-surgery (Day 2), and 7 days post-surgery (Day 8). All three evaluations included assessments of muscle strength and power, as well as performance-based function, hip pain, and thigh swelling. In addition, blood sampling to assess systemic inflammation was conducted Pre-surgery and on Day 2, and self-reported disability was assessed Pre-surgery and on Day 8. All measurements were performed and recorded by the same experienced investigator, who was blinded to the results of all previous visits. The reporting of the study follows the recommendations of The Strengthening the Reporting of Observational Studies in Epidemiology (STROBE, [please see Supporting Information]) [31].

### Participants

Between March and December 2011, patients awaiting a THA were included by convenience sampling from the pre-surgery program at the Department of Orthopedic Surgery at Copenhagen University Hospital, Hvidovre, Denmark, if they were one of the first three patients each day to receive a THA. To ensure sufficient reliability of the applied measurements, the same experienced investigator was used, which is why it was only possible to include a maximum of three patients per day. To reduce the risk of selection bias, the person responsible for assigning the patients' surgical times was kept unaware of the current study.

The inclusion criteria were: planned primary, unilateral THA due to end-stage osteoarthritis and age >18 years. The exclusion criteria were: inability to speak or understand Danish, inability to perform the outcome measures due to other diseases, such as rheumatoid arthritis, polyneuropathy, or extremity peripheral paresis.

### Procedures

#### Peri-operative care

All patients were operated on using the posterior approach (standard, no minimally invasive surgery) with an uncemented THA. They followed a fast-track program for THA, which has been described in detail elsewhere [32]. Briefly, the program included standardized preoperative multidisciplinary education, multi-modal pain treatment for 6 days (a daily dose of gabapentin (900 mg/day), paracetamol (4 g/day), and celecoxib (400 mg/day) initiated on the morning of surgery, and continued for 6 days with morphine (10 mg) as a rescue analgesic for moderate or severe pain), and postoperative rehabilitation, including early ambulation but no progressive strength training. All patients were discharged to their homes, according to well-defined functional discharge criteria [33]. The length of hospital stay was counted as the number of nights hospitalized after the operation. Patients were told to continue with further daily analgesics and follow the 6-day prescribed pain treatment ordered by their general practitioner.

#### Outcome measures

Muscle strength of the hip actions: adduction, abduction and flexion in the operated leg was measured during isometric contractions, using a hand-held dynamometer (Chiroform ApS, Viborg, Denmark), according to Thorborg and co-workers [34]. After warm up and familiarization with the procedure, the muscle strength of hip flexion in sitting position, and hip abduction and adduction in the supine position, was determined. The hip action-order was randomized, and the order was the same for each patient for all evaluations. The patients performed four maximal contractions – separated by 60-sec pauses – and the contraction that elicited the largest force was used as the data point. Standardized verbal encouragement was provided during each trial (contraction). Muscle strength was subsequently expressed as maximal voluntary torque per kilogram of body mass (Nm/kg). The external lever-arm for hip flexion was the distance from the anterior superior iliac spine to 5 cm proximal to the patella border, for hip abduction it was the distance from the anterior superior iliac spine to 5 cm proximal to the proximal edge of the lateral malleolus, and for hip adduction it was the distance from the anterior superior iliac spine to 5 cm proximal to the proximal edge of the medial malleolus.

Leg-press power of the operated leg was estimated from a rapid extension of the operated leg from a flexed position, using a Nottingham Power Rig (Medical Physics and Clinical Engineering Department, Nottingham, United Kingdom), according to Short & Bassey [35]. Positioning of the patient was as follows: each patient sat in the rig with the knee and hip flexed in a self-selected comfortable position with the foot of the leg to be tested on the pedal, and secured on the seat with a seatbelt across the hips. The patient was then instructed to extend the leg to press the pedal as quickly as possible with maximum effort, with 60-sec pauses between trials. Standardized verbal encouragement was provided during each trial. The test was ended when leg press power decreased in two consecutive trials or ten trials had been performed. The press that had the highest power was identified, and power per kilogram of body mass was then used as the data point. For all evaluations, the patients used their available range of knee motion during the presses.

Performance-based function was assessed using The Timed Up & Go (TUG) [36] and 10-meter fast speed walking [37] tests, by measuring the time (in seconds using a stopwatch) required to rise from a chair, walk 3 meters, and return to the chair (TUG), and walk 10 meters as quickly and safely as possible (10-meter fast speed walking), using the habitual walking aid, if any was used. The trial that required the least number of seconds was used as the data point for each of the two performance-based functions. For details, see [22].

Hip pain was quantified at rest (supine), before the experimental protocol, and activity, using a 100-mm Visual Analog Scale (VAS), with endpoints – visible to the patients – of “no pain” (0) and “worst pain imaginable” (100). Activity-based recordings of hip pain were made during the tests of muscle strength/power and performance-based function, before which the patients were told to register hip pain during test performance and then rate it immediately after test termination. The pain recordings made during the trials that were subsequently selected as muscle strength, power, or performance-based function data points, were selected as the pain data points.

Self-reported disability was assessed using The Hip Disability and Osteoarthritis Outcome Score (HOOS) [38]. It is a 40-item questionnaire; constructed to assess patient-relevant outcomes in five separate subscales (Pain, Symptoms, Activities of daily living (ADL), Sport and recreational activities (Sport/rec), and Hip-related quality of life (QOL). For each subscale, a normalized score was then calculated with 100 indicating no symptoms and 0 indicating extreme symptoms.

Thigh volume was used as a surrogate measure of fluid accumulation around the operated hip joint, as intra-articular swelling could not be assessed, and because thigh swelling has been found to relate to knee-extension strength soon after hip fracture surgery [39]. Thigh volume was estimated using tape-measured circumferences of the upper and lower thigh with the patient lying in the supine position, using the Fustrum method [40]. It is based on the assumption that the thigh is approximately in the shape of a truncated cone, or frustum. The upper circumference was measured 25 cm proximal to the border of the patella, and the lower circumference, 5 cm proximal to the border of the patella. For details, see [40].

Systemic inflammation was measured by C-Reactive Protein (CRP) in venous blood preoperatively, and 24 hours after surgical incision. Concentrations of CRP were measured in blood samples drawn in 4 ml tubes with gel and lithium-heparin using a COBAS 6000 analyzer (Roche Diagnostics, Mannheim, Germany).

### Statistical analyses

All variables, except hip pain, were normally distributed (Kolmogorov-Smirnoff). Normally distributed data are reported as means±1 standard deviation (SD), and non-normally distributed data as medians with inter-quartile ranges. Data from a pilot study indicated substantial strength, power, and functional performance losses at Days 2 and 8. Hence, the current study was powered (80% power) to detect changes of 30% or more for all strength, power and functional performance measurements, based on the measurement that required the greatest sample, which was 30 patients. These analyses were considered primary and powered to the primary objective of the study, while all secondary (correlation) analyses pertaining to the secondary objective of the study, were considered exploratory. A significance level of 5% (*P*<0.05) was used.

Changes in muscle strength and thigh volume over time (Pre-surgery, Day 2 and Day 8) were investigated using one-way repeated measures analyses of variance (ANOVAs). If a significant main effect of time was determined, pair-wise Bonferroni-corrected comparisons were used to identify differences between time points. Performance-based function are reported using descriptive statistics only, as a change in walking aid occurred between time points. Changes in hip pain over time (Pre-surgery, Day 2 and Day 8) were investigated using Friedman's Two-Way Analyses of Variance by Ranks. If a significant main effect of time was determined, Wilcoxońs Signed Rank tests with Bonferroni corrections were used to identify differences between time points. Changes in self-reported disability (HOOS) and systemic inflammation from Pre-surgery were investigated using paired-samples *t* tests. Between-variable correlations were quantified using Pearson or Spearman correlation coefficients, depending on normal distribution or not. All analyses were performed for the 30 patients, who completed all follow-up visits (per-protocol-analyses).

## Results

### Participants

As the study used convenience sampling, eligibility was only examined on the days available for experimental work. Hence, the number of potentially eligible patients is unknown. The number of patients examined for eligibility was 40. Of these, 5 were excluded: 3 declined to participate, 1 had the surgery postponed due to suspicion of spinal disc herniation, and 1 had a planned bilateral, simultaneous THA instead of a unilateral THA. The number of patients confirmed eligible was 35, and these were included. Five dropped out after inclusion: three on post-operative Day 1 (1 due to hip dislocation twelve hours post-surgery, 1 due to proximal femoral fracture and hip dislocation two hours post surgery, 1 due to personal reasons), two between Day 2 and Day 8 (1 due to observation for acute myocardial infarction, 1 due to a hip fracture). The baseline characteristics of the remaining 30 patients, who attended all follow-up visits and had complete data sets, are shown in Table 1.

**Table 1:** Patient characteristics (n = 30).

Category	Result
Age, yrs	65.9±7.2
Women, number (%)	21 (70)
Height, cm	169.8±8.4
Body Mass, kg	81.0±17.1
Body Mass Index, kg/m^2^	28.0±4.7
Length of stay*	1.9±1.0

Values are means±1SD, unless otherwise indicated. *postoperative nights.

### Muscle strength and power

**Figure 1:**
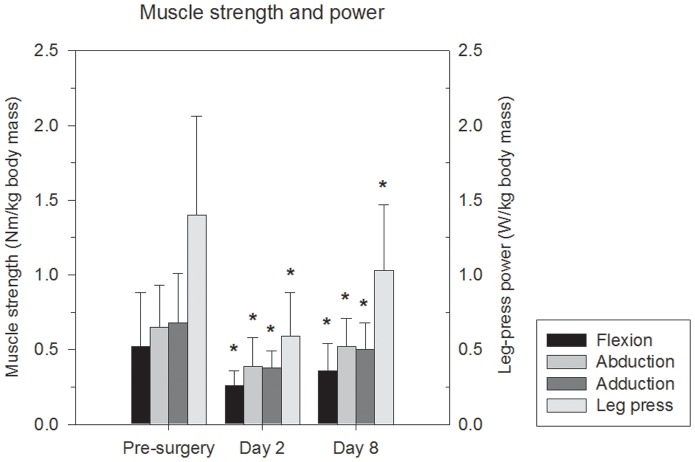
Changes in muscle strength/power over time in the operated leg. The secondary y-axis applies to the leg-press power measurement. *Day 2 statistically different from Pre-surgery, and Day 8 statistically different from Day 2 and Pre-surgery.

Changes in muscle strength and power are shown in Figure 1. Hip flexion, abduction, adduction, and leg-press power changed significantly over time, as ANOVA main effects were observed (*P*<0.017). The subsequent multiple comparisons revealed that muscle strength at Day 2 was reduced compared to Pre-surgery (hip flexion, abduction, adduction, and leg-press power *P*<0.001), that muscle strength at Day 8 was reduced compared to Pre-surgery (hip flexion, abduction, adduction, and leg-press power *P*<0.017), and that muscle strength increased from Day 2 to Day 8 (hip flexion, abduction, adduction, and leg-press power *P*<0.006). The muscle strength and power losses at Day 2 varied between 41% and 58%, with the greatest loss observed for leg-press power. The strength and power losses at Day 8 varied between 23% and 31%, with the greatest loss observed for hip-flexion strength. No correlations between hip muscle strength or leg press power and hip pain, reported during the tests of muscle strength and power, were observed.

### Performance-based function

Performance-based function, assessed by the TUG and 10-meter fast speed walking tests, was indicated to have changed significantly over time, based on the descriptive statistics. Pre-surgery, where no patients used walking aids, the mean TUG-performance was 9.5±2.7 s, at Day 2, where all patients walked on two crutches, the mean TUG-performance was 18.3±6.5 s, and at Day 8, where 7 patients walked without walking aids, 10 walked with one crutch, and 13 with two crutches, the mean TUG-performance was 11.7±3.4 s. For the 10-meter fast speed walking test, the patients used the same walking aids as described above. The mean 10-meter fast speed walking test-performance was 1.40±0.31 m/s, 0.84±0.24 m/s, 1.14±0.32 m/s, at Pre-surgery, Day 2, and Day 8, respectively.

### Self-reported disability

**Figure 2:**
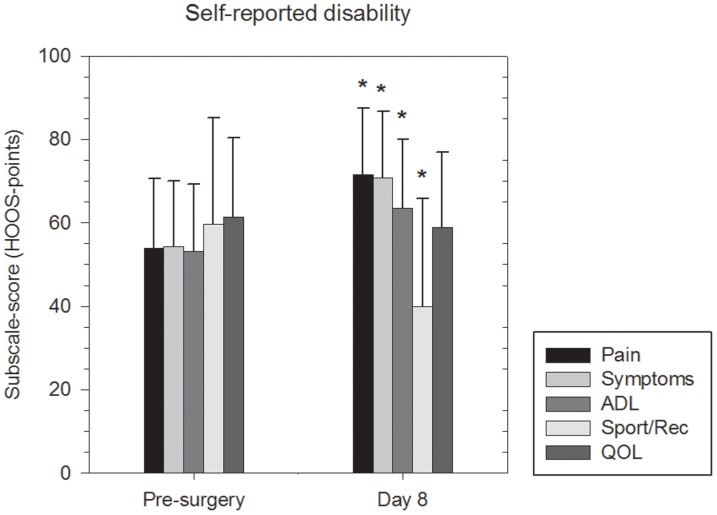
Changes in self-reported disability over time in the operated leg. *statistically different from Pre-surgery.

Self-reported disability, assessed by the HOOS, is shown in Figure 2. It improved from Pre-surgery to Day 8 for the subscales: Pain (pre: 53.9±16.6, post: 71.5±15.9, *P*<0.001), Symptoms (pre: 54.3±15.7, post: 70.8±15.8, *P*<0.001), ADL (pre: 53.1±16.2, post: 63.5±16.5, *P*<0.014), whereas it decreased for the subscale Sport/rec (pre: 59.6±25.5, post: 40.4±25.8, *P*<0.002) and did not change for QOL (pre: 61.5±19.0, post: 59.0±17.9, *P* = 0.679).

### Thigh volume, hip pain and systemic inflammation

Generally, thigh volume increased over time (Pre-surgery: 4167.1±808.2 cm^3^, Day 2: 4376.1±834.1 cm^3^, Day 8: 4489.1±861.8 cm^3^), whereas hip pain at rest, maximal contractions, and performance-based function decreased over time, from mild to moderate Pre-surgery to mild at Days 2 and 8 (Table 2). Systemic inflammation (CRP) increased from 2.9±4.2 mg/l Pre-surgery to 84.3±35.0 mg/l at Day 2 (*P*<0.001). The increases in thigh volume and systemic inflammation did not correlate with any of the observed lower extremity strength or power losses.

**Table 2:** Changes in hip pain at rest, and during maximal contractions and performance-based function.

Category	Pre-surgery	Day 2	Day 8
	(n = 30)	(n = 30)	(n = 30)
Resting pain, VAS-mm	10.0 (2.3–18.3)	11 (0–22.0)	1.5 (0–9.0)*‡$
Hip-flexion MVC pain, VAS-mm	21 (4.8–39.0)	21 (10.0–38.5)	14.5 (4.0–22.3)
Hip-abduction MVC pain, VAS-mm	20 (4.8–40.0)	20.5 (8.8–40.0)	10.5 (3.0–27.3)*‡$
Hip-adduction MVC pain, VAS-mm	18.5 (3.0–31.3)	21.5 (11.5–41.3)	8.5 (3.8–17.3)*$
Leg-press power pain, VAS-mm	23.5 (5.0–34.3)	20.0 (6.8–42.0)	10 (2.3–18.0)*‡$
“Timed Up & Go” pain, VAS-mm	21 (5.5–30.0)	15.5 (5.0–30.3)	5.0 (0–23.5)*‡$
“10 m walk” pain, VAS-mm	20.5 (6.0–40.0)	20.0 (3.8–33.0)	5.5 (1.5–18.3)*‡$

*Main effect of time, ‡Day 8 significantly different from Pre-surgery, and $Day 8 significantly different from Day 2. Data are medians (inter-quartile range). MVC, Maximal voluntary contraction; VAS, Visual Analog Scale

## Discussion

The main findings of the current study were (i) considerable loss of hip muscle strength and leg-press power postoperatively at Day 2, (ii) this loss was reduced but still lower at Day 8 compared with Pre-surgery, and (iii) changes in hip pain, thigh swelling or systemic inflammation were not associated with loss of hip muscle strength or leg-press power.

### Loss of muscle strength and power after THA

The reasons for aiming to reach preoperative muscle strength values and, preferably, that of age-matched, healthy peers after THA are several. Firstly, muscle strength of the operated leg relates to functional performance soon after THA as well as much later [20]. Secondly, lower-extremity muscle weakness of the operated leg and asymmetric leg loading during sit-to-stands (healthy leg compensation) have been reported years after THA [41], [42]. Finally, muscle strength decreases with increasing age [43]. Hence, surgery-induced reductions in muscle strength that are not addressed, may, theoretically, precipitate a loss of functional independence just a few years later. This is especially the case where strength values after surgery are close to cut points [44] for losing functional independence because of insufficient lower-extremity muscle strength.

Loss of hip muscle strength and leg-press power have been reported previously, at later time points – weeks to months – after surgery [4], [5], [8], [14]–[19], [22]. The current data more or less reflect surgery-induced muscle strength/power loss with spontaneous recovery soon after surgery, as previously no progressive strength training of these muscle groups had been instituted as early as this at our institution. The losses were pronounced at Day 2, but were generally returning towards values observed Pre-surgery. Acknowledging that values Pre-surgery may be reduced compared to those of age-matched, healthy peers due to end-stage osteoarthritis [4], [5], it still indicates substantial spontaneous recovery of muscle strength in the first week after THA, which is in contrast to that observed after total knee arthroplasty [45], [46]. One reason for this may be that the patients included in the current study entered a well-established fast-track post-surgery program [33], focusing on enhancing recovery by, for example, mobilization with full weight-bearing allowed within only a few hours of surgery. The use of opioid-sparing multimodal pain treatment – resulting in relatively little postoperative pain, nausea, and vomiting – also allows for earlier and maybe more extensive mobilization and time out of bed. These factors could explain the reduced losses.

The apparent rapid rate of recovery of muscle strength within the first week is supported by previous work, in which hip and knee muscle strength 8 weeks after standard THA did not differ from pre-operative values [5]. However, authors have reported persisting hip-muscle atrophy [30] and strength deficits years after surgery, when compared to that of either the contra-lateral side [5], [14], or age-matched peers [16], [17], indicating a need for more intense types of physical rehabilitation. In many cases, the intensity of the rehabilitation provided after surgery seems sub-optimal, i.e. with too little intensity [23]. Pertaining to this notion, there are indications that both muscle strength and performance-based function recover more quickly after supervised rehabilitation introduced soon after surgery, including progressive strength training, compared to a lesser intensive, supervised rehabilitation control [21], [29]. It therefore seems very relevant to investigate – on a larger scale – if the apparent rate of early recovery of muscle strength and functional performance following the operation can be accelerated further by a brief period of intensive and well-described rehabilitation introduced soon after surgery, including progressive strength training [47] of the hip muscles and leg extensors. Prior to this, it may be especially important to identify patients, who do not display the same rate of recovery, and hence, require more prolonged rehabilitation. It could be the case for patients with low(er) preoperative strength and functional performance [6]. Likewise, there may also be a proportion of patients with high(er) preoperative strength and functional performance, who require less or no supervised rehabilitation.

Acknowledging that the secondary analyses may have been underpowered (see methodological limitations), the current data do not lend much support for thigh swelling, systemic inflammation, or hip pain as candidates, underlying the initial losses of muscle strength after THA. However, failure of the central nervous system to activate muscles close to the operated hip joint due to intra-articular swelling warrants future investigation, as reduced neuromuscular activity of the hip muscles has been reported in healthy subjects after intra-articular injections [26]. The role of the inflammatory response for multi-level strength-losses after THA also warrants future investigation, as it has been indicated to affect early functional recovery (walking) after THA [48]. Post-operative hip pain, however, seems clinically insignificant. The reported pain intensities at Days 2 and 8 during rest, maximal contractions, and performance-based function were low and decreased over time, compared to Pre-surgery.

### Loss of functional performance after THA

Loss of performance-based function soon after THA has been reported previously [22]. The current data seem to concur with this. We were only able to perform descriptive analyses of performance based function, as walking aids were self-selected and, thus, not controlled for. The reason for this was that we did not find it feasible to control for, this early after surgery. With this limitation in mind, performance-based function seems to reach preoperative values around 6–9 weeks after surgery [6], whereas self-reported function (WOMAC physical function) seems to reach preoperative values 1–2 weeks after surgery [6], depending on the applied outcome measure and type of post-operative rehabilitation. In the current study, self-reported function (HOOS ADL) improved from Pre-surgery to Day 8, while performance-based function was still markedly reduced. This discrepancy in the time courses of performance-based and self-reported function after THA is also observed in total knee arthroplasty [49], [50]. The results underscore the importance of having both a performance-based and self-reported measure of function with acceptable psychometric properties, when quantifying changes in functional performance over time after THA, as these measures seem to capture different aspects of recovery after THA.

Generally, given the early loss of muscle strength and power, it is suggested that rehabilitation soon after THA should include progressive strength training [47] of both hip flexors, hip adductors, hip abductors, and leg extensors, as progressive strength training increases muscle strength of the operated leg when initiated early after THA [11], [12], [21], and appears safe [11], [12], [21]. Leg presses seem especially important, and they will also, to some degree, involve the hip adductors and abductors, in addition to all the leg extensors.

### Study limitations

A few precautions should be made when interpreting the current data. Firstly, the study used a design with convenience sampling, which may, unintentionally, cause some selection bias. It was only possible to include a maximum of three patients per day, on days available for experimental work. However, the person responsible for assigning the patients' surgical times was kept unaware of the current study and, hence, did not know the distribution of experimental days, and that the maximum inclusion per experimental day was three patients. Secondly, the study was powered with respect to changes in muscle strength, power, and functional performance. All correlation analyses relating to the secondary objective were exploratory, which is why potential causes of the initial muscle strength loss and power loss may not have been indicated. Thirdly, as previously mentioned, some patients changed habitual walking aids during the course of the study, which may – in itself – have had an impact on their performance-based function. This is difficult to control for, so we performed these analyses using descriptive statistics only. Nevertheless, despite these limitations, the main finding of substantial strength loss and indications of performance-based function loss soon after THA in the current study is supported by previous related findings at later time points, which strengthens the external validity of these findings.

## Conclusion

Hip muscle strength and leg-press power, decreased substantially in the first week after THA – especially at Day 2 – with some recovery evident at Day 8. In contrast, self-reported symptoms and function improved. Hip pain, thigh swelling, and systemic inflammation were not related to the observed muscle strength loss and power loss. These data on surgery-induced changes in lower extremity muscle strength may help in the design of impairment-directed, postoperative rehabilitation soon after THA, by serving as an indicator of muscle strength status prior to out-patient physical rehabilitation.

## Supporting Information

Checklist S1 **STROBE checklist of items that should be included in reports of cohort studies.**
(PDF)
